# Benzoic Acid-Inducible Gene Expression in Mycobacteria

**DOI:** 10.1371/journal.pone.0134544

**Published:** 2015-09-08

**Authors:** Marte S. Dragset, Amy K. Barczak, Nisha Kannan, Mali Mærk, Trude H. Flo, Svein Valla, Eric J. Rubin, Magnus Steigedal

**Affiliations:** 1 Department of Immunology and Infectious Diseases, Harvard School of Public Health, Boston, Massachusetts, United States of America; 2 Centre of Molecular Inflammation Research, Department of Cancer Research and Molecular Medicine, Norwegian University of Science and Technology, Trondheim, Norway; 3 Department of Biotechnology, Norwegian University of Science and Technology, Trondheim, Norway; 4 Massachusetts General Hospital, Department of Medicine, Boston, Massachusetts, United States of America; 5 Central Norway Regional Health Authority, Stjørdal, Norway; Centre National de la Recherche Scientifique, FRANCE

## Abstract

Conditional expression is a powerful tool to investigate the role of bacterial genes. Here, we adapt the *Pseudomonas putida*-derived positively regulated XylS/*Pm* expression system to control inducible gene expression in *Mycobacterium smegmatis* and *Mycobacterium tuberculosis*, the causative agent of human tuberculosis. By making simple changes to a Gram-negative broad-host-range XylS/*Pm-*regulated gene expression vector, we prove that it is possible to adapt this well-studied expression system to non-Gram-negative species. With the benzoic acid-derived inducer *m*-toluate, we achieve a robust, time- and dose-dependent reversible induction of *Pm*-mediated expression in mycobacteria, with low background expression levels. XylS/*Pm* is thus an important addition to existing mycobacterial expression tools, especially when low basal expression is of particular importance.

## Introduction

Tuberculosis (TB), caused by *Mycobacterium tuberculosis* (*Mtb*), continues to be one of the most serious lethal infectious diseases in the world. The World Health Organization reports around 9 million new cases and 1.5 million deaths annually as a result of TB [1]. The spread of drug-resistant mycobacteria adds to the importance of developing new anti-mycobacterial therapies [2]. To improve TB therapeutics, it is crucial to better understand the underlying molecular mechanisms of the disease, and to gain new insights it is important to create a greater selection of new and enhanced molecular tools.

Inducible expression systems have become fundamental engines for genetic research. For instance, over-expression or conditional regulation of genes enables us to map gene function, determine gene essentiality and validate drug targets [3–5]. Regulators of inducible expression systems can be divided into three groups; activators (positive regulation), repressors (negative regulation) and regulators with dual activity (positive and negative regulation) [6]. Upon induction, activators commonly lead to activation of transcription by interacting with the RNA polymerase and thereby increase the affinity between the polymerase and the promoter. Conversely, a repressor will typically bind to the operator sequence in the absence of inducer and inhibit transcription initiation, for instance by preventing the binding of RNA polymerase to the promoter. Thus, the steady state level of the repressor in the various bacterial growth phases is important to keep basal expression low. In the presence of inducer the repressor undergoes conformational changes, making it unable to bind the operator and thus allow transcription [6, 7]. Regulators with dual activity can act as repressors or activators of the same promoter, depending on presence or absence of inducer [8]. While the majority of inducible expression systems are negatively regulated, it has been proposed that positively regulated systems, independent of repressor activity, are better candidates when tight control with low basal expression levels is desired [9, 10].

In addition to low basal expression, a good mycobacterial inducible expression system should provide robust and reversible induction by an inexpensive, non-toxic inducer which penetrates cells by diffusion, induce a dose- and time-dependent response and have the potential to work across mycobacterial species as well as in macrophage and animal infection models.

While the lack of a diverse collection of mycobacterial inducible expression tools has hampered research for decades, widely used inducible expression systems were developed for *Escherichia coli* by the early 1980s [11, 12]. The first equivalent system in mycobacteria, the acetamide-inducible system based on the *Mycobacterium smegmatis* (*Msmeg*) endogenous acetamidase promoter [13], was developed as late as in 1997. A few years later, a temperature-inducible expression cassette, based on the *Ptra* promoter and a derivative of the TraR repressor, was demonstrated to be functional but weak in mycobacteria [14]. More recently, several tetracycline (tet) repressor-based inducible systems have been developed [15–18], together with pristinamycin, nitril, arabinose and IPTG-inducible expression tools [19–22]. The most recent contribution to mycobacterial inducible expression is a riboswitch-based theophylline responsive system regulated at a translational level [23]. The aforementioned systems have various strengths and weaknesses as expression tools; all are however dependent on repression to lower expression in the uninduced state. The nitril-inducible NitR/*PnitA* system adapted from the related Gram-positive *Rhodococcus rhodochrous* is controlled by the AraC-family regulator NitR proposed to work as both a repressor and activator, albeit the dual function of NitR has not yet been experimentally verified [19, 24]. Thus, developing a positively regulated system independent of repression of the inducible promoter could greatly benefit mycobacterial research.

XylS/*Pm* is a well-studied, positively regulated expression system derived from the *Pseudomonas putida* TOL plasmid pWWO [25], and it is known to function in a wide range of Gram-negative bacterial species [26]. The inducible *Pm* promoter originates from the transcriptional regulation of the *meta*-cleavage pathway for benzoate and alkylbenzoate catabolism controlled by the activator XylS and is to our knowledge fully independent of transcriptional repression in basal expression mode [6]. Upon induction by benzoate-derived effectors, such as *m*-toluate, XylS dimerizes and binds to the *Pm* promoter where it interacts with the transcription initiation machinery and activates transcription [27, 28].

In this study we adapt XylS/*Pm* for use in *Mtb* and *Msmeg*, introducing the first positively regulated expression system to mycobacteria. We show robust time- and dose-dependent inducible expression from the *Pm* promoter, with a basal expression comparably lower to commonly used mycobacterial expression tools. Importantly, by showing the function of XylS/*Pm*-controlled expression in non-Gram-negative species, we demonstrate the great potential of expanding the use of this expression system to other Gram-positive bacteria.

## Materials and Methods

### Bacterial strains and culture conditions

*Msmeg* mc^2^155 [29], *Msmeg* DM22 [30] (kindly provided by Kurt Krause, University of Otago, New Zealand) and *Mtb* H37Rv (ATCC 25618) were cultured in Middlebrook 7H9 (BD Difco) supplemented with 0.2% glycerol, 0.05% Tween80 and 10% ADC (*Msmeg*: 50 g BSA fraction V, 20 g dextrose, 8.5 g NaCl, 0.03 g catalase, dH_2_O up to 1 L) or OADC (*Mtb*: BD Difco). Kanamycin was added to 20 μg/ml in mycobacteria and to 50 μg/ml in *E*. *coli*. Hygromycin was added to 50 μg/ml in mycobacteria. Colonies of *Msmeg* were grown on LB agar plates, whereas colonies of *Mtb* were grown on 7H10 (BD Difco) supplemented with glycerol and 10% OADC (BD Difco). Standard cloning and mycobacterial transformation techniques were used.

### Determination of *Mtb* growth in *m*-toluate

Strain H37Rv was grown to mid log phase in standard 7H9 media. The culture was then diluted to an OD_600_ of 0.01 and aliquoted in a 96-well plate with the final concentrations of *m*-toluate as indicated in the respective figure. Ethanol carrier concentration was equal in all conditions. Plates were incubated at 37°C for 14 days. Cultures were then resuspended well, and OD_600_ was determined using a Spectramax 5 (Molecular Devices).

### Expression vector construction

To create the expression plasmid pMDX, a DNA fragment containing a) *xylS* controlled by *P*_*myc1*_ *tetO* promoter [16] (called *Ptet* in this study), b) reverse TetR#28 [31] (called reverse TetR in this study) controlled by *Psmyc* promoter [16], c) *Pm* promoter and d) transcriptional terminators strategically placed was synthesized by Genscript USA Inc (NJ, USA). The *xylS* and *Pm* sequences were obtained from pKT1 [32]. The synthetic expression cassette described above (GenBank accession number KT239651) was cloned into the backbone of pMV261 [33] using Not*I*. pMDX-luc was created by PCR amplification of the firefly luciferase gene from pMH109 (kindly provided by Mark Hickey/David Sherman, Seattle Biomed, Seattle, WA, USA) [34, 35], using primers LucMDX_F and LucMDX_R. pMDX-zeo was created by PCR amplification of the zeocin resistance gene *Sh ble* (GenBank accession number ABW35374.1) from pER10 (unpublished data) using primers ZeoMDX_F and ZeoMDX_R. The amplified genes were separately cloned into pMDX using the Eco*RI* and Nde*I* sites, placing the reporter genes under control of the *Pm* promoter. pTET-zeo was created by PCR amplification of the zeocin gene from pMDX-zeo using the primer pair ZeoPacIF and ZeoPstIR. The amplified gene was cloned into the Pac*I* and Pst*I* sites of pUV15TetORm [16]. pMDX-hyg-alr was constructed by amplifying the hygromycin resistance cassette from *Msmeg* Δ*fxbA* strain [36] using primers pMDXHyG-F-NheI and pMDXHyG-R-SpeI and clone it into Nhe*I* and Spe*I* restriction sites of pMDX, before the complete alanine racemase (*alr*) gene was amplified from *Msmeg* mc^2^155 [29] with primers pMDXAlr-F-NdeI and pMDXAlr-R- EcoRI and cloned into Nde*I* and Eco*RI* restriction sites of pMDX. The pTET-alr was constructed by amplifying the alanine racemase gene from Msmeg mc^2^155 [29] using primers pTetOAlr-F-Pac I and pTetOAlr-R-Sbf I and clone it into Pac*I* and Sbf*I* restriction sites of pTET-zeo. pMDXint and pMDXint-luc was created by amplifying integrase and mycobacteriophage L5 attP-site from pMC1s [16] using primers pMC1_F and pMC1_R. The generated PCR product was digested with Pci*I* and Eco*RI* and cloned into the Pci*I* and Eco*RI* sites of pMDX and pMDX-luc replacing pAL5000, creating pMDXint and pMDXint-luc, respectively. pUV15TetORm and pMC1s were kindly provided by Sabine Ehrt/Dirk Schnappinger, Weill Cornell Medical College, New York, USA. Details of the plasmids and primer sequences can be found in Table 1 and Table 2, respectively.

**Table 1:** Plasmids used in this study.

Plasmid	Properties relevant to study	Source
pMDX	Kan^r^, XylS controlled by *Ptet*, reverse TetR controlled by *Psmyc*, *Pm*, *oriM* (pAL5000), *oriE* (pBR322).	This study
pMDX-luc	pMDX derivative. Firefly luciferase gene from pMH109 cloned under control of Pm.	This study
pMDX-zeo	pMDX derivative. *Sh ble* gene cloned under control of *Pm*.	This study
pMDXint	Kan^r^, XylS under control of *Ptet*, reverse TetR controlled by *Psmyc*, *Pm*, integrase, attP-site	This study
pMDXint-luc	pMDXint derivative, *Pm* controlling firefly luciferase gene from pMH109, integrase, attP-site.	This study
pMC1s	Integrase, attP-site	[16]
pNBV1	Hyg^r^, *E*. *coli*—mycobacterial shuttle vector	[37]
pKT1	Kan^r^, XylS controlled by *Ps2*, firefly luciferase gene from *Photinus pyralis* controlled by *Pm*, *oriE* (RK2).	[32]
pMH109	Hyg^r^, attP-site, firefly luciferase gene controlled by MOP.	[34, 35]
pER10	Zeo^r^ (*Sh ble* gene: GenBank accession number ABW35374.1)	unpublished data
pMV261	Kan^r^, *oriM* (pAL5000), *oriE* (pBR322).	[33]
pTET-zeo	Hyg^r^, *P*_*myc1*_ *tetO* in control of *Sh ble*. TetR.	This study
pUV15TetORm	Hyg^r^, Kan^r^, *P*_*myc1*_ *tetO* in control of GFP. Tn10 derived TetR controlled by *Pimyc*. *oriM* (pAL5000), *oriE* (pBR322).	[16]
pUV15tetORm::luciferase	Derivative of pUV15TetORm. *P*_*myc1*_ *tetO* in control of luciferase.	[38]
pMDX-hyg-alr	pMDX derivative. *alr* gene cloned under control of *Pm*. Hyg^r^ added.	This study
pTET-alr	pTET-zeo derivative. *P*_*myc1*_ *tetO* in control of *alr*.	This study

Kan^r^: kanamycin resistance. Hyg^r^: hygromycin resistance. Zeo^r^: zeocin resistance.

**Table 2:** Oligonucleotides used in this study.

Primer	Sequence, 5’-3’
LucMDX_F	AGTCATGAACATATGGAAGACGCCAAAAACAT
LucMDX_R	ATCGAATTCTCTAGAATTACACGGCGATCTTTCC
ZeoMDX_F	AGTCATGAACATATGGCCAAGTTGACCAGTGCCG
ZeoMDX_R	ATCGAATTCTCTAGAATCAGTCCTGCTCCTCGGCCACGA
ZeoPacIF	CGCATGCTTAATTAAGAAGGAGATATACATATGGCCAAGTTGACCAGTGC
ZeoPstIR	CCTCCTACTGCAGCCCGGGGGAGCCATTTAAATGCTCGAATTCATCAGTCCTGCTCCTCGGCCA
pMC1_F	TTGCTGATACATGTGGAACGGCAATGCTCCTGG
pMC1_R	GGATCCAGCTGCAGAATTCG
pMDXHyG-F-NheI	CTAGCTAGCCTGACGTTCATCCATAGT
pMDXHyG-R-SpeI	CACTAGTGCGGCTTTAGCTAATTAA
pMDXAlr-F- NdeI	CCATATGCAGACCACCGAGCCCATG
pMDXAlr-R- EcoRI	GGAATTCTCAATCTTGTTGTCCTGCGC
pTetOAlr-F-Pac I	CCTTAATTAA ATGCAGACCACCGAGCCC
pTetOAlr-R-Sbf I	ATTGGTTCCTGCAGGTCAATCTTGTTGTCCTGCGC

### XylS/*Pm* induction protocol

*Msmeg* or *Mtb* transformed with either pMDX (negative control), pMDX-luc, pMDX-zeo, pMDXint, pMDXint-luc or pMH109 (positive control for luciferase assays) were grown in 7H9 with supplements and 20 μg/ml kanamycin to stationary phase, then diluted approximately 1:500 and grown to OD_600_ 0.05–0.1 at 37°C. At this point, the samples were normalized to identical optical densities (ODs) and induced with the indicated amount of *m*-toluate (Sigma, 1 M stock solution solved in laboratory grade ethanol). For uninduced controls the same amount of ethanol was added in place of inducer. The samples were incubated with shaking at 30°C or 37°C for the number of hours specified.

### *Mtb* tetracycline-inducible luciferase comparison

*Mtb* H37Rv containing pMDX-luc or pUV15tetORm::luciferase (a gift of Deborah Hung, The Broad Institute [38]) was grown to mid log phase, and then diluted to an OD_600_ of 0.01. For pMDX-luc strains, *m*-toluate or an equivalent amount of ethanol carrier was added to each roller bottle. For pUV15tetORm::luciferase strains, anhydrous tetracycline or an equivalent amount of water carrier was added to each roller bottle. Cultures were grown with rolling at 37°C. At the indicated time points, aliquots were taken for luciferase assays as described.

### Luciferase reporter gene assays

After induction, OD_600_ was measured and noted. Cells were spun down at 4000 rpm for 10 minutes and pellets completely dried for any excess media before being resuspended in 100 μl dH_2_O. 30 μl of the resuspended cells were mixed with 7 μl 5x Passive Lysis Buffer (Promega) in the appropriate 96-well plate and incubated with shaking for 30 minutes at 37°C for sufficient lysis. After incubation, assay substrate (Promega, E1500) was prepared according to manufacturer’s instructions and 75 μl added to each well containing cell lysis mixture. Bioluminescence was subsequently measured according to manufacturer’s recommendations on a Fluoroskan Ascent FL (Labsystems). The relative luminescence units (RLU) were recorded and normalized with regard to OD_600_ or colony forming units (CFU). To measure the amount of luciferase produced, cell pellets from uninduced or induced cultures were resuspended in 1 ml Passive Lysis Buffer and the cells were disrupted using 0.1 mm glass beads and an MP FastPrep-24 (3 x 45 sec, 6000 M/S). The samples were normalized by OD before cell disruption. 50 μl cell lysate was combined with 50 μl luciferase assay substrate and luciferase activity was measured as above. The amount of luciferase produced was determined by comparison to known luciferase concentrations (luciferase obtained from Abcam) and normalized to total protein levels as measured by BioRad Protein Assay.

### Zeo^r^ (*Sh ble*) gene assay

On solid media: after 5 hours pre-induction with 1.5 mM *m*-toluate (induced) or ethanol (uninduced), *Msmeg* cultures containing were normalized by OD measurements and serially diluted. The dilutions were spotted on LB agar plates with 20 μg/ml kanamycin and increasing amounts of zeocin (Invitrogen). Induced samples were spotted on plates containing 1.5 mM *m*-toluate, and uninduced samples were spotted on plates containing ethanol instead of *m*-toluate. The plates were incubated at 30°C for 2 days.

In liquid media: after 5 hours pre-induction with 1.5 mM *m*-toluate, induced and uninduced *Msmeg* cultures were normalized to OD_600_ 0.005 and grown in the presence of increasing concentrations of zeocin and 1.5 mM *m*-toluate (induced) or ethanol (uninduced) in special micro-plate “honeycomb” wells (Oy Growth Curves Ab Ltd). Growth was monitored by Bioscreen (Oy Growth Curves Ab Ltd), registering OD_600_ every 2 hours. For the comparison between pMDX-zeo and pTET-zeo, the cultures were diluted directly to OD_600_ 0.005, without pre-induction. The pMDX-zeo strain was grown in the presence or absence of 1.5 mM *m*-toluate, while the pTET-zeo strain was grown in the presence or absence of 200 ng/ml anhydro-tetracycline (atc, Sigma).

## Results

### Functional adaptation of XylS/*Pm* expression system for regulation of gene expression in mycobacteria

We wanted to investigate whether the Gram-negative, positive regulator system XylS/*Pm* could be functional in mycobacteria. We determined that the most direct way to evaluate the induction potential of this system would be by simply cloning the entire Gram-negative broad host range expression cassette into a mycobacteria-*E*. *coli* shuttle vector, introducing it into mycobacteria and assessing for inducibility. Hence, we took the XylS/*Pm* cassette from pKT1 [32], cloning XylS with its native constitutive promoter and the reporter gene encoding firefly luciferase expressed from the inducible *Pm* promoter, into the shuttle vector pNBV1 [37]. However, no induction of luciferase expression was observed in *Msmeg*, despite optimization of induction time, inducer concentration and growth phase. We could, on the other hand, observe low basal expression from *Pm* independent of presence or absence of inducer (results not shown). Clearly, several factors could explain the lack of induction; the inducer might be unable to diffuse through the complex mycobacterial cell wall, XylS might not be properly expressed, the RNA polymerase might not interact with XylS or the *Pm* promoter and/or the chosen reporter gene might not be properly transcribed or translated in mycobacteria. Finally, the reporter gene product could be toxic to the new host cell when over-produced. To unravel potential obstacles, we tested whether the inducer could enter the cell, hypothesizing that increased concentrations of inducer would be toxic if successfully entering the bacteria. *Msmeg* was grown in the presence of increasing amounts of *m*-toluate, and indeed, we saw increasing toxicity proportional to increasing inducer concentration (S1 Fig indicating that the inducer enters the cells.

We then changed the native promoter driving expression of *xylS* to a mycobacterial promoter, ensuring proper constitutive expression of the activator. We chose the *P*_*myc1*_ *tetO* promoter (called *Ptet* in our study) from Ehrt *et al*.’s adapted tet-inducible system, based on its constitutive function in the absence of the TetR repressor [16]. Excess of XylS has previously been shown to stimulate expression from *Pm* in the absence of inducer [28, 39, 40]. To overcome the potential increase in background expression due to over-production of XylS, we introduced a mechanism for turning off *xylS* transcription by adding a mutant version of TetR to our construct. In contrast to standard TetR, the mutant acts as a reverse repressor, binding rather than releasing the operators in *Ptet* upon tetracycline induction (TetR#28 from [31]). In the event of high basal expression due to excessive XylS, we could tune down XylS production by adding anhydro-tetracycline (atc) to the media, facilitating tighter regulatory control and lower levels of basal expression.

**Fig 1:**
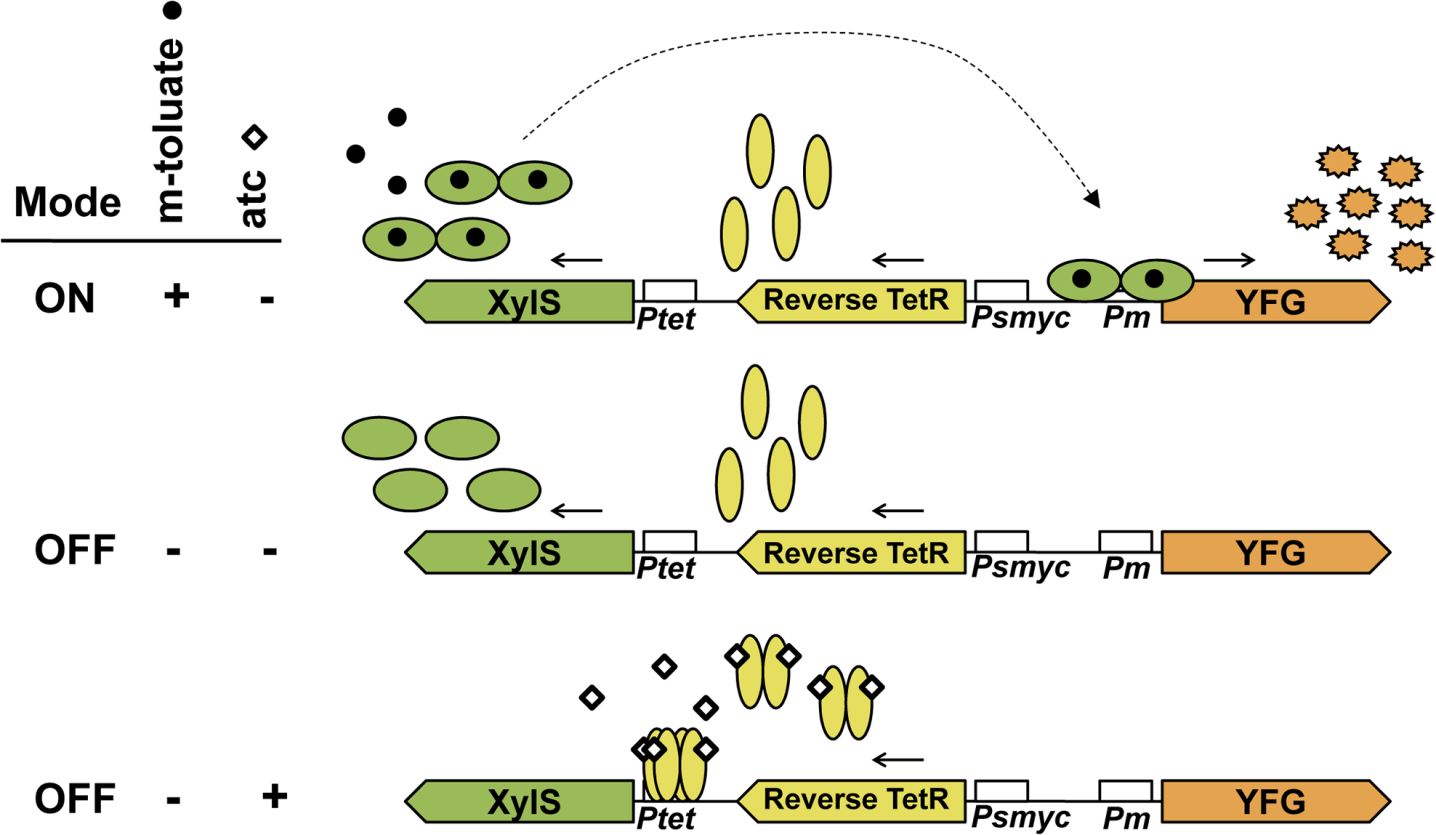
Benzoic acid-inducible expression system, XylS/*Pm*, for regulation of genes in mycobacteria. The inducible *Pm* promoter and its activator XylS regulate the expression of “your favorite gene” (YFG). XylS is constitutively expressed under control of *Ptet* in the absence of anhydro-tetracycline (atc) and binds the *Pm* promoter in the presence of the inducer *m*-toluate. This facilitates expression of YFG and leaves the expression system ON (upper panel). In the absence of *m*-toluate, XylS is not activated, leaving the expression system OFF, as expression from *Pm* is not induced (middle panel). Reverse TetR is constitutively expressed by *Psmyc*, and binds the operator in *Ptet* in the presence of atc blocking transcription of *xylS*. Addition of atc leaves the system in a more fully OFF mode as potential basal *Pm*-mediated transcription caused by excessive levels of XylS is abolished (lower panel).

To address the possibility of luciferase reporter toxicity to our cells, the firefly luciferase reporter gene was changed from wild type to a modified version of the firefly luciferase previously shown to be functional in mycobacteria [34, 35]. As presented in Fig 1, the adapted XylS/*Pm* expression system is in an OFF mode at baseline (no induced transcription from *Pm*) in the absence of the inducer *m*-toluate (Fig 1, middle panel). However, to potentially further minimize background expression, atc can be added to repress *xylS* transcription, leaving the system in a more fully OFF mode (Fig 1, lower panel). Transcription from *Pm* is fully ON in the presence of *m*-toluate and in the absence of tetracycline (Fig 1, upper panel).

### Induction of *Pm* with *m*-toluate is robust, time- and dose-dependent in mycobacteria

**Fig 2:**
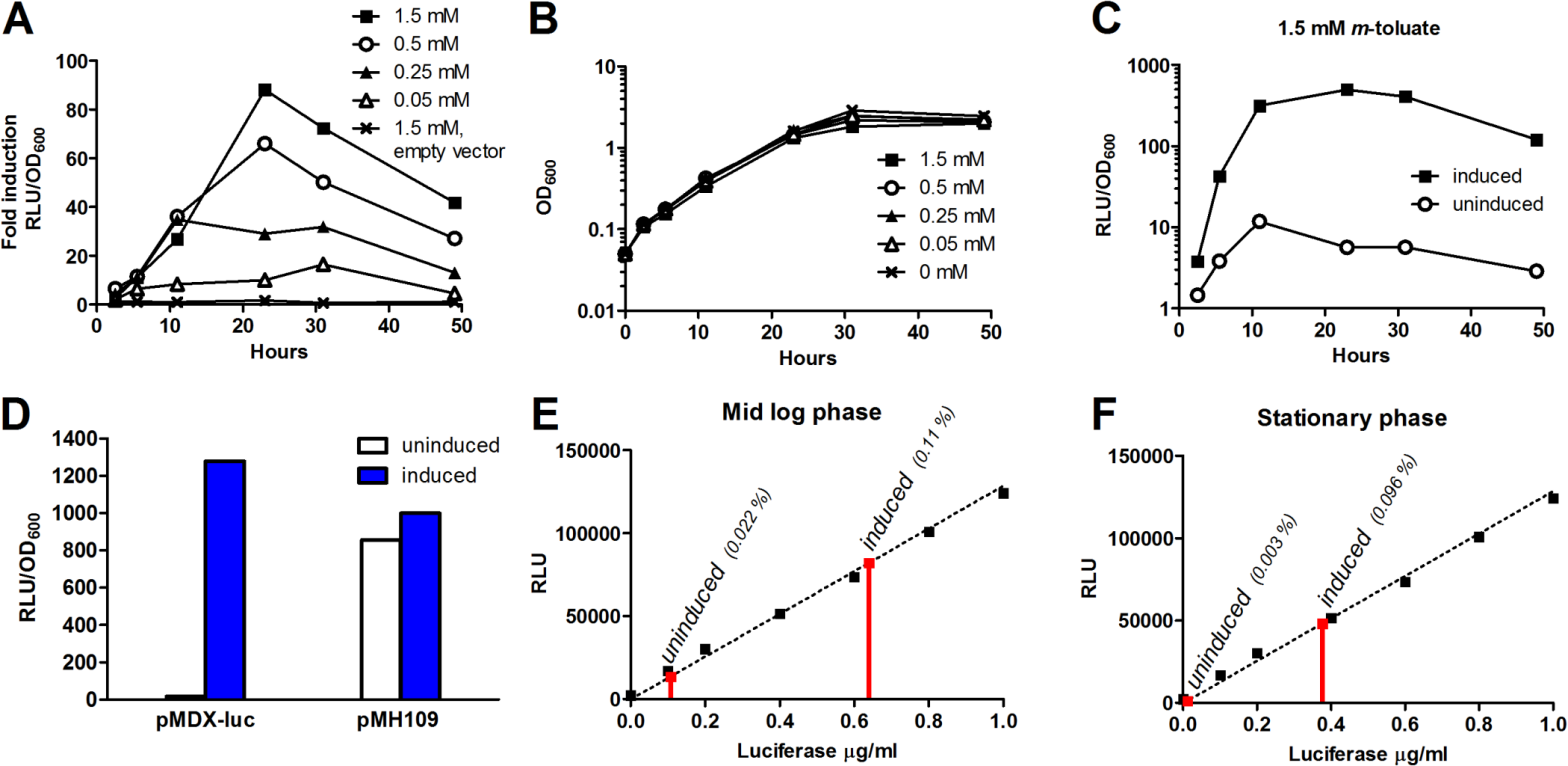
Induction of *Pm* with *m*-toluate is robust, time- and dose-dependent. *Msmeg* transformed with the expression vectors pMDX-luc or the empty vector pMDX (no reporter gene) treated with increasing concentrations of *m*-toluate (induced) or ethanol carrier (uninduced). Cells were incubated at 30°C, and luciferase expression was determined at 2.5, 5.5, 11, 23, 31 and 49 hours after addition of *m*-toluate. (A) Fold induction of RLU in induced samples compared to uninduced samples. (B) Growth of uninduced and induced samples of pMDX-luc corresponding to samples in (A). (C) Time course of luciferase induction from pMDX-luc with 1.5 mM *m*-toluate or ethanol carrier. (D) Maximal induction of pMH109 and pMDX-luc-transformed *Msmeg* induced with 2 mM *m*-toluate. (E) Amount of luciferase produced as determined by the activity of known luciferase concentrations (0, 0.1, 0.2, 0.4 0.6, 0.8 and 1 μg/ml luciferase) in mid log phase or (F) stationary phase. Luciferase fraction of total bacterial protein shown in brackets. RLUs were normalized to the OD_600_ of the samples before luciferase assay. All results are representative of two or more independent experiments.

Following modification of the XylS/*Pm* expression cassette, we examined whether gene expression could, in fact, be induced in mycobacteria by addition of *m*-toluate. We introduced our adapted expression system with luciferase as a reporter gene (pMDX-luc) to *Msmeg*, and by optimizing inducer concentration and induction time we were able to achieve robust induction. When adding 1.5 mM *m*-toluate, we reached up to 90-fold induction (ratio of induced to uninduced expression levels) during late log phase (Fig 2A and 2B). Furthermore, we demonstrated that induction was time-dependent, peaking after 24 hours, and dose-dependent, rising nearly proportionally with increased inducer concentrations. In contrast, uninduced background levels of luciferase did not increase over time (Fig 2A and 2C). Normalizing luciferase expression to CFU instead of OD_600_ gave an enhanced induction pattern after 24 hours; we reached 240-fold induction by CFU normalization (S2 Fig).

The amount of luciferase expressed was determined by measuring relative luminesecence units, RLUs, which are relative. To get a sense of expression from *Pm* relative to other standard mycobacterial promoters, we compared our induced levels of luciferase with the stably expressed levels from the strong constitutive mycobacterial optimal promoter, MOP [35]. We transformed *Msmeg* with pMH109, an integrative plasmid containing our luciferase gene under control of MOP [35]. As seen in Fig 2D, when *Pm* was induced with 2 mM *m*-toluate, luciferase expression exceeded the level expressed from MOP, indicating that a high level of expression can be achieved using XylS/*Pm*. Furthermore, to approximate the amount of luciferase produced, sample luciferase activity was compared to the activity of known luciferase concentrations. Fig 2E and 2F show the level of luciferase produced for uninduced and induced samples at mid log and stationary phase, respectively. When normalized to the total protein concentration of the lysed cell samples, the amount of luciferase constituted approximately 0.022% (0.1 μg/ml luciferase) and 0.003% (0.012 μg/ml luciferase) of total protein in uninduced samples in mid log and stationary phase, respectively. Induced samples contained 0.11% (mid log, 0.64 μg/ml) and 0.096% (stationary, 0.38 μg/ml) luciferase.

Our pMDX-luc plasmid is episomal (pAL5000 ori) with an estimated copy number of 3–10 in mycobacteria [41]. To determine whether we could also induce expression from a chromosomally integrated single copy of the XylS/*Pm* expression cassette, we constructed pMDXint-luc, a vector integrating at the mycobacteriophage L5 attachment site. Again using luciferase as the reporter gene, we induced pMDXint-luc-transformed *Msmeg* alongside our *Msmeg* pMDX-luc strain, demonstrating inducible expression (~10-fold induction) even when the expression system was chromosomally integrated (S3 Fig).

Our initial *Msmeg* induction experiments presented in Fig 2A–2D were carried out at 30°C, based on favorable conditions for soluble recombinant protein production in Gram-negatives [42] and standard protocols for XylS/*Pm* expression in Gram-negatives [43]. Although our adapted version of XylS/*Pm* works well in *Msmeg* at 30°C, we wanted to compare induction at different temperatures to determine the system’s utility in standard conditions used for mycobacterial experiments. Hence, we induced our *Msmeg* pMDX-luc strain with different concentrations of *m*-toluate and incubated the samples at either 30°C or 37°C during induction. *Pm* was clearly induced at 37°C, reaching even higher induction ratios than at 30°C (S4 Fig).

**Fig 3:**
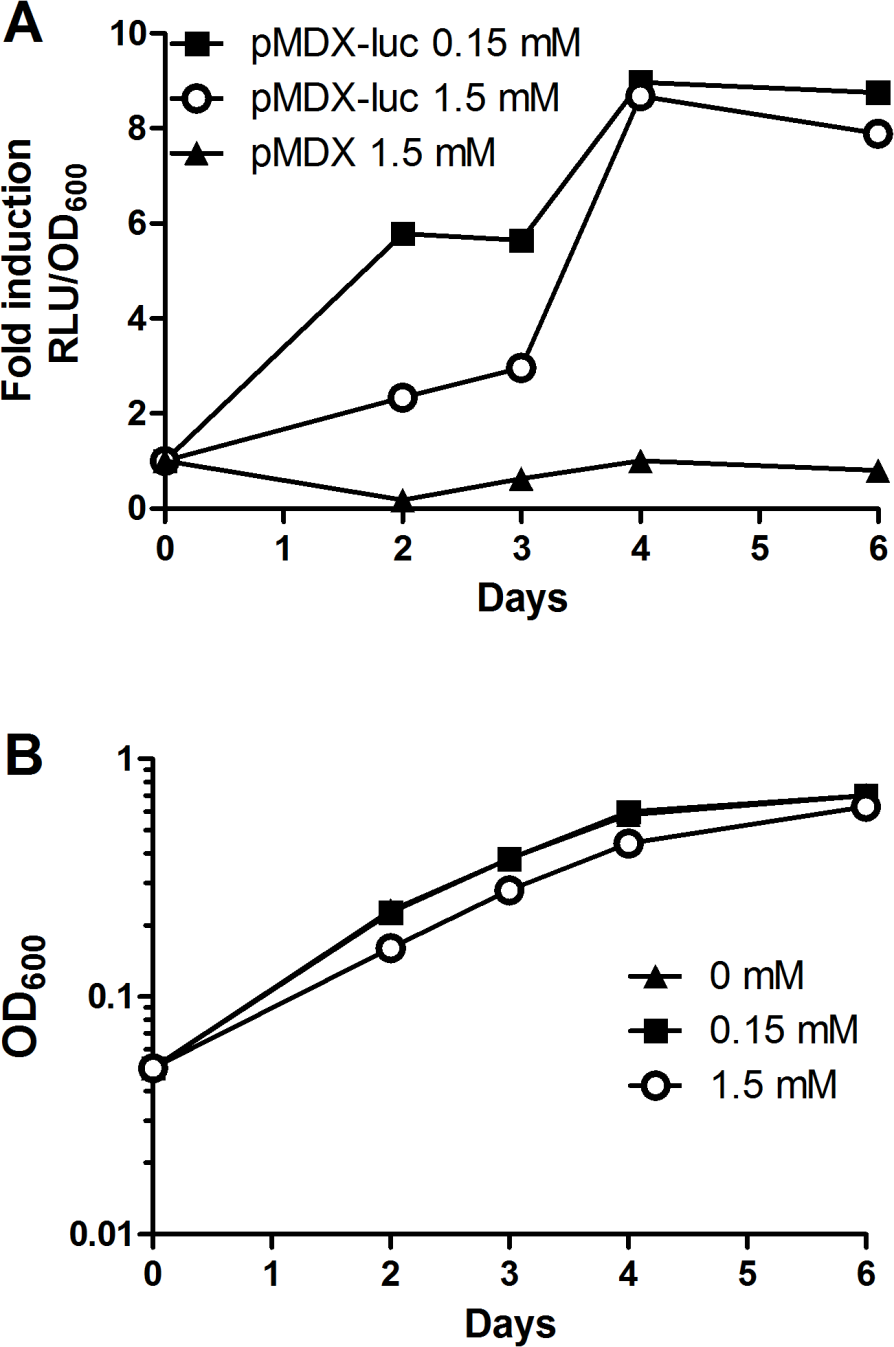
Induction of *Pm* in *Mtb*. *Mtb* transformed with the expression vector pMDX-luc or the empty vector pMDX were induced with indicated concentrations of *m*-toluate (induced), or ethanol carrier (uninduced). Samples were maintained rolling at 37°C and analyzed for luciferase expression levels at 2, 3, 4 and 6 days after induction. (A) Fold induction of RLU of induced to uninduced samples normalized for OD_600_. (B) Growth of uninduced and induced samples containing pMDX-luc corresponding to samples in (A). The results are representative for two independent experiments.

After optimizing induction in *Msmeg*, we turned to *Mtb* to see if we could induce expression through *Pm* in a comparable fashion in the pathogenic species. pMDX-luc-transformed *Mtb* was induced in a similar manner as described for *Msmeg*. As shown in Fig 3A, the *Pm* promoter was clearly inducible in *Mtb*, and showed a time-dependent response peaking in late log phase. Maximum induced expression was overall lower in *Mtb* than in *Msmeg* (Fig 3A and 3B). *Mtb* showed a similar tolerance pattern towards *m*-toluate as *Msmeg* (S1 Fig). Taken together, our results show that we are able to achieve a robust, time- and dose-dependent induction of the *Pm*-promoter in mycobacteria.

### Addition of anhydro-tetracycline restricts *Pm*-mediated induction through inhibition of *xylS* expression

**Fig 4:**
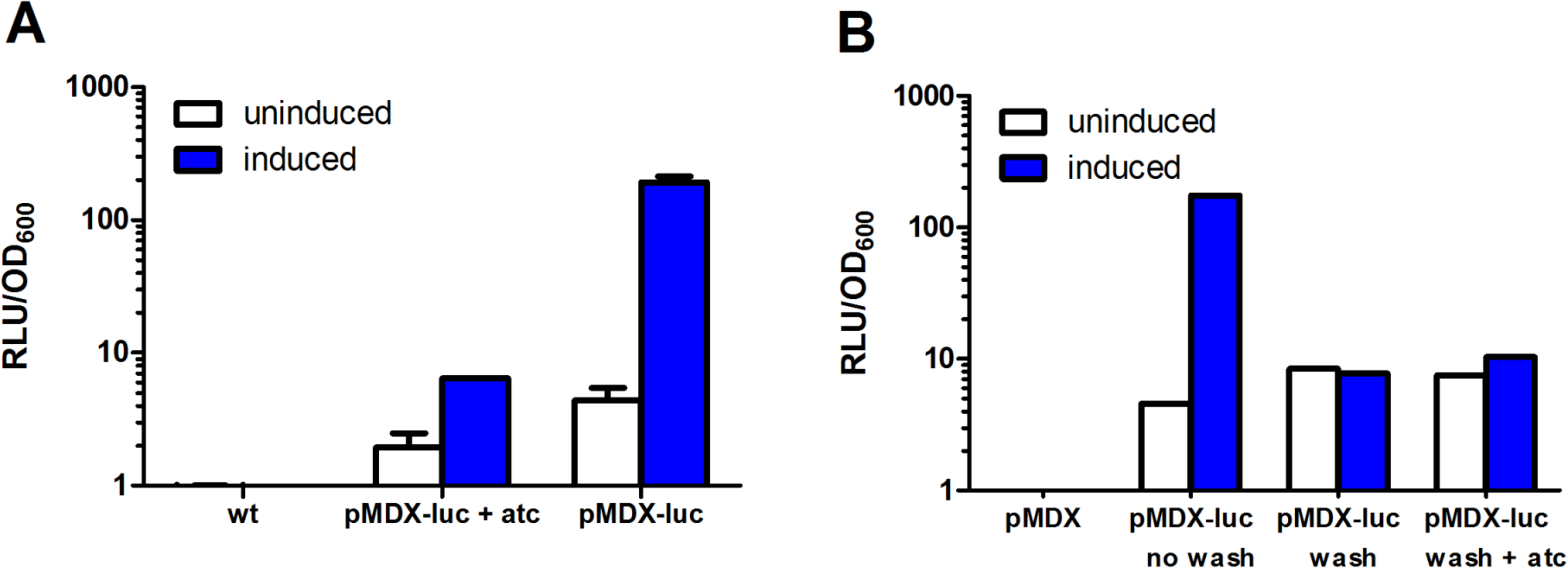
Induced expression from *Pm* is reversible. (A) *Msmeg* transformed with pMDX-luc were grown to OD_600_ 0.05–0.1 in the presence of anhydro-tetracycline (+ atc), or in the absence of atc. Cells were then induced with 1.5 mM *m*-toluate for 24 hours and grown with shaking at 30°C before RLU was measured. *Msmeg* wild type was included as negative control. Error bars represent standard deviations. (B) *Msmeg* pMDX-luc was induced to maximum fold induction (~90 fold—not shown). Cells were then either washed once in 7H9 (wash), or left untreated (no wash) before diluted to OD_600_ 0.05–0.1. Washed samples were grown to late log phase in the presence (+ atc) or absence of atc. Unwashed samples were grown to late log phase in the absence of atc. Cells transformed with pMDX (empty vector) were included as negative control. RLUs were normalized by OD_600_. The results are representative for two or more independent experiments.

Positively regulated expression systems are proposed to have lower basal transcription rates than negatively regulated systems [9, 10]. We wanted to investigate whether repressing XylS production would decrease the basal expression from *Pm* even further. First, to confirm that adding anhydro-tetracycline (atc) to the media would activate the reverse TetR to bind the operator and reduce transcription of the *xylS* gene, we incubated our *Msmeg* pMDX-luc strain with or without 100 ng/ml atc over night, thus giving TetR time to bind and inhibit *Ptet*-controlled *xylS*-transcription. Aside from addition of atc, the samples were treated identically, and induced to maximum expression of luciferase (Fig 4A). We could clearly see that the addition of atc markedly reduced expression of luciferase from the *Pm* promoter, most likely due to the lack of activator in the cells. However, inhibiting *xylS* expression by atc did not seem to further lower the basal expression from *Pm* (Fig 4A), suggesting that background expression is likely to be mediated by *Msmeg* endogenous transcription machinery rather than excessive XylS production.

### Induced expression from *Pm* can be reversed by inducer washout

Mapping gene function and essentiality are important aspects of mycobacterial research. To investigate phenotypes arising from very low expression levels of the genes of interest, being able to reverse induced expression is crucial. To investigate whether removing the inducer by washout could reduce expression from *Pm* in mycobacteria, we induced our *Msmeg* pMDX-luc strain to maximum luciferase expression (about 90-fold, results not shown) before washing the cells in inducer-free media upon subcultivation or directly subcultivation without washing. When the subcultures reached late log phase, we measured luciferase activity (Fig 4B). After subcultivation, only the unwashed sample showed induction (about 40 fold), demonstrating that inducer washout before re-cultivation is enough to completely return the cells to an uninduced state.

We furthermore investigated whether a combination of inducer washout and addition of atc to turn off *xylS* expression would lower the basal *Pm* expression. We added 100 ng/ml atc to washed cells upon subcultivation, and allowed them grow to late log phase in the presence of atc before measuring luciferase. Compared with wash alone, no further decrease in background expression levels was observed in the presence of atc (Fig 4B). Together, our results demonstrate that we could successfully revert to uninduced levels of expression by inducer washout prior to subcultivation.

### Regulation of the zeocin resistance gene (*Sh ble*) by XylS/*Pm*

**Fig 5:**
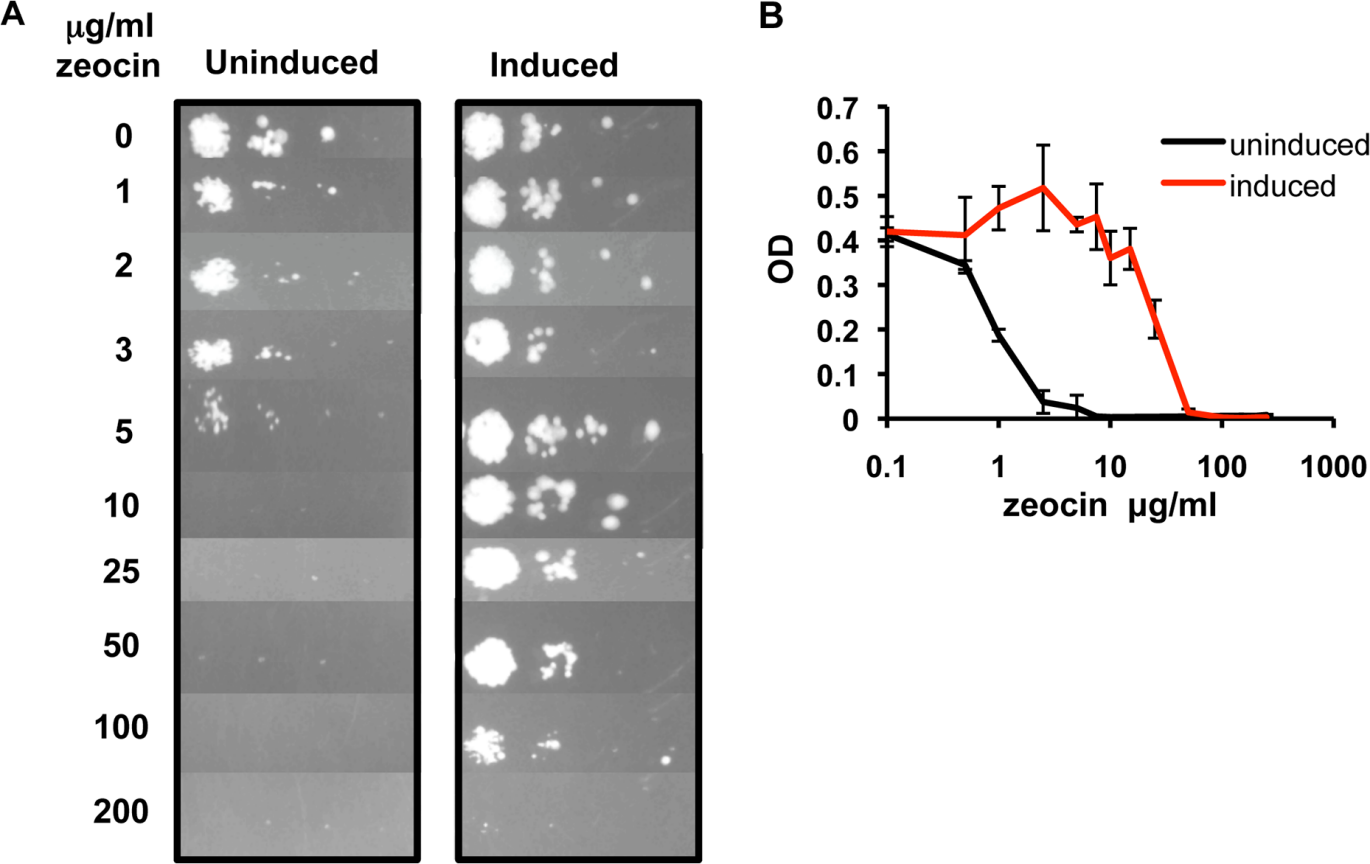
Regulation of the zeocin resistance gene in the presence of zeocin. (A) *Msmeg* transformed with pMDX-zeo was grown to OD_600_ 0.05–0.1 before addition of 1.5 mM *m*-toluate (induced) or ethanol carrier (uninduced) was added. Cells were then incubated for 5 hours at 30°C. The cells were normalized by OD_600_, serially diluted, and spotted on plates containing increasing amounts of zeocin and 1.5 mM *m*-toluate (induced) or ethanol (uninduced) and incubated at 30°C for 2 days. (B) *Msmeg* transformed with pMDX-zeo was pre-induced for 5 hours as described above, then diluted to OD_600_ 0.005 and grown in triplicates in micro-plate wells in the presence of increasing concentrations of zeocin (0, 0.5, 2.5, 5, 7.5, 10, 15, 25, 50, 100, 150, 200 and 250 μg/ml) and 1.5 mM *m*-toluate (induced) or ethanol carrier (uninduced) shaking at 37°C. Growth was monitored by Bioscreen, registering OD_600_ every other hour. The samples are presented by the OD_600_ of uninduced or induced *Msmeg* pMDX-zeo in increasing concentrations of zeocin, when the respective sample grown in the *absence* of zeocin reached mid log phase. Error bars represent standard deviations and the results represent three independent experiments.

After demonstrating that we could induce gene expression via *Pm* in a precisely controlled fashion, we wanted to investigate whether *Pm* could be used for conditional regulation of biologically active genes in mycobacteria. Zeocin is an antibiotic of the bleomycin/phleomycin family that causes cell death by cleaving DNA. Resistance to zeocin is conferred by the *Sh ble* gene encoding a small acidic protein, which inhibits the antibiotic by binding to it in a stoichiometric manner [44]. By taking advantage of these properties we can mimic the regulation of a conditional essential gene in mycobacteria, making the *Sh ble* gene essential for survival in the presence of zeocin. Hence, the *Sh ble* gene was cloned under control of XylS/*Pm*, creating pMDX-zeo. *Msmeg* transformed with pMDX-zeo was induced for 5 hours to give the *Sh ble* gene a chance to be expressed at low levels. The culture was then serially diluted and spotted on agar plates containing both *m*-toluate and increasing concentrations of zeocin (Fig 5A); plates were then incubated for 48 hours. Uninduced controls were included in the experiment by simultaneous spotting on plates lacking *m*-toluate. As shown in Fig 5A, the initial number of bacteria seen on the uninduced and induced plates were identical (compare uninduced and induced samples, 0 μg/ml zeocin). However, induced cells could survive concentrations of zeocin up to 100–200 μg/ml due to the elevated production of the conditionally essential *Sh ble* gene product; a few small colonies were still visible in induced sample at the 200 μg/ml concentration. Moreover, the smaller colony sizes seen in the uninduced sample in the presence of zeocin concentrations as low as 1 μg/ml suggest that the uninduced cells have limited ability to grow under even low levels of zeocin stress (Fig 5A).

To get a more detailed picture of the zeocin-mediated growth inhibition, we diluted pre-induced cultures of *Msmeg* pMDX-zeo in increasing concentrations of zeocin with or without *m*-toluate. OD_600_ was measured every two hours over a 48 hour period, and the absorbance measured for cells grown in increasing zeocin concentrations was determined at the time point the sample, induced or uninduced, grown in *absence* of zeocin had reached mid log phase (Fig 5B). Uninduced sample showed a significantly reduced growth rate in zeocin concentrations as low as 1 μg/ml, and growth was nearly completely inhibited at 2.5 μg/ml. Induced samples grew close to normally with up to 15 μg/ml zeocin before the growth rate was gradually reduced. Controls with empty vector lacking *Sh ble* grew only in media without zeocin (results not shown). These results show that the XylS/*Pm* expression system could serve as a tool to regulate conditionally essential genes in mycobacteria.

### *Pm* compares favorably to *Ptet* in basal expression levels in *Msmeg*

So far, we have established XylS/*Pm* as a functional expression tool in *Msmeg* and *Mtb*, and demonstrated that it is a potential tool for conditional control of essential genes in mycobacteria. We wanted to investigate next how XylS/*Pm* compared to a commonly used inducible mycobacterial expression tool, with emphasis on basal expression levels. We cloned the *Sh ble* gene conferring resistance to zeocin under control of *Ptet* in pUV15TetORm (Tn*10*-derived TetR controlled by the intermediate strong promoter *Pimyc* and pAL5000 mycobacterial ori) [16] for direct comparison to XylS/*Pm*, creating pTET-zeo. The *Pm* and *Ptet*-regulated systems were introduced to *Msmeg*, induced and monitored for 120 hours in increasing concentrations of zeocin (S5 Fig).

**Fig 6:**
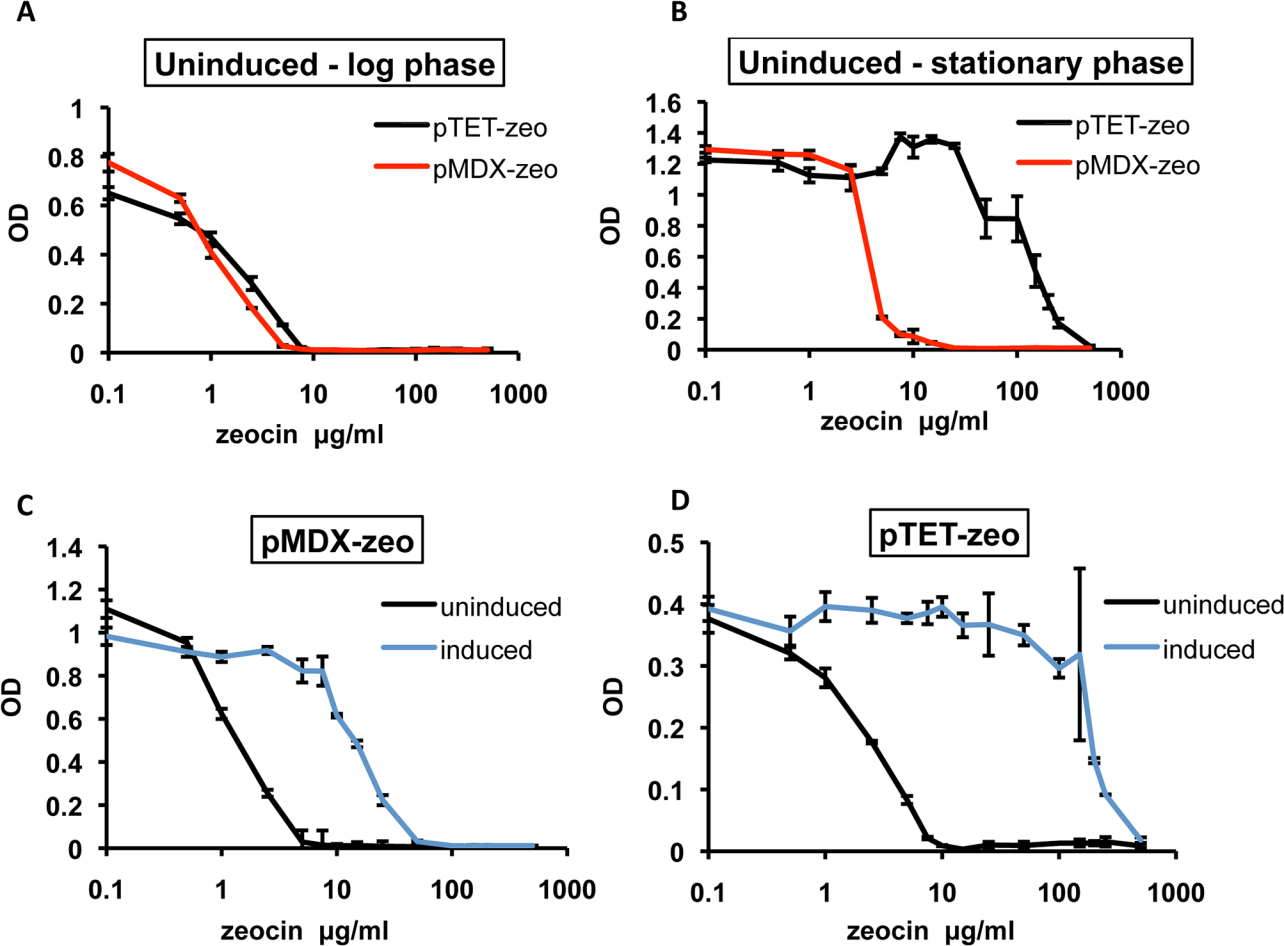
*Pm*–mediated basal expression is low and compares favorably to *Ptet*-mediated basal expression in *Msmeg*. (A-B) *Msmeg* transformed with pMDX-zeo or pTET-zeo was diluted to OD_600_ 0.005 in the presence or absence of m-toluate (1.5 mM) or atc (200 ng/ml), respectively, and increasing amounts of zeocin (0, 0.5, 2.5, 5, 7.5, 10, 15, 25, 50, 100, 150, 200, 250 or 500 μg/ml). Samples were grown in triplicates and monitored for 120 hours by a Bioscreen, registering OD_600_ every other hour. Basal expression from *Pm* and *Ptet* is presented by growth of *Msmeg* pMDX-zeo and *Msmeg* pTET-zeo in increasing concentrations of zeocin, when the respective sample grown in the *absence* of zeocin reached mid log phase (A) or stationary phase (B). (C) Induced and uninduced samples of pMDX-zeo strain in late log phase. (D) Induced and uninduced samples of pTET-zeo strain in mid log phase. The results represent two independent experiments.

*Pm* and *Ptet*-mediated basal expression was compared through determination of the amount of zeocin tolerated by the uninduced expression strains in log phase or stationary phase. While we observed comparable sensitivity to increasing concentrations of zeocin in uninduced *Pm* and *Ptet*-regulated strains in log phase (Fig 6A), the *Ptet*-regulated strain could grow in the presence of higher concentrations of zeocin than the *Pm*-regulated strain in stationary phase in the absence of inducer (Fig 6B). The pMDX-zeo strain was severely growth restricted in the presence of as little as 5 μg/ml zeocin, whereas the pTET-zeo strain could tolerate up to 250 μg/ml zeocin before reaching the same level of growth inhibition in stationary phase (Fig 6B). These results suggest that basal expression from *Ptet* generates significantly higher levels of *Sh ble* transcripts than *Pm* during prolonged growth.

Comparison of induced samples of pMDX-zeo and pTET-zeo revealed higher levels of *Sh ble* expression in *Ptet* than *Pm*-regulated cells. pTET-zeo cultures grew nearly unaffected by the antibiotic up to 50 μg/ml zeocin in mid log phase (under optimized conditions for tet-inducible expression [16]) (Fig 6D), while growth of pMDX-zeo cultures was markedly inhibited by 10 μg/ml zeocin under optimized conditions (Fig 6C). Overall, *Ptet*-regulated cells showed around 10-fold higher induced levels of the zeocin resistance protein compared to *Pm*.

Although *Pm* drives lower levels of basal expression than commonly used inducible promoter systems during extended growth, *Pm*-mediated background expression might still be of biological relevance. In S6 Fig we cloned the alanine racemase gene (*alr*), which gene product catalyzes the conversion of L-alanine to D-alanine, under the control of XylS/*Pm*. For this particular gene, the background levels of *alr* expression were sufficient for *Msmeg* to produce enough D-alanine for normal growth. The same was seen for *Ptet-*regulated *alr* expression, and similar results were obtained for both *Ptet* and *Pm*-regulated *alr* expression on solid (S6 Fig) and liquid media (results not shown). These results underline that successful regulation of conditionally essential genes using inducible expression systems depends largely on the gene in question.

### *Pm* compares favorably to *Ptet* in basal expression levels in *Mtb*

**Fig 7:**
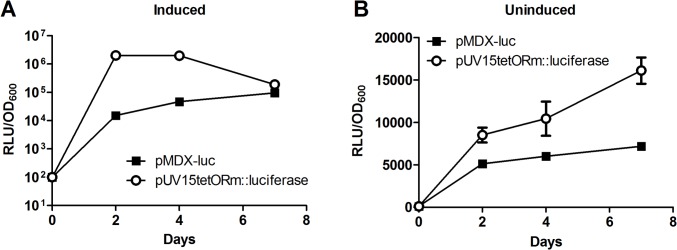
*Pm*–mediated basal expression is low and compares favorably to *Ptet-*mediated basal expression in *Mtb*. (A-B) *Mtb* transformed with pMDX-luc or pUV15tetORm::luciferase was diluted to OD_600_ 0.005 in the presence or absence of m-toluate (1.5 mM) or atc (200 ng/ml), respectively. Samples were grown in triplicates and monitored for 7 days registering OD_600_ at 2, 4 and 7 days. Basal expression from *Pm* and *Ptet* is presented by level of luciferase produced by *Mtb* pMDX-luc and *Mtb* pUV15tetORm::luciferase. (A) shows the luciferase expression over time in induced sample and (B) shows the basal expression from uninduced samples over time. The results represent two independent experiments.

The behavior of *Pm* in *Mtb* was investigated by transformation of pMDX-luc or pUV15tetORm::luciferase, and comparing *Pm* and *Ptet*-mediated basal expression through determination of the levels of luciferase produced by the two strains under induced and uninduced conditions. As seen for *Sh ble*, pUV15tetORm::luciferase revealed higher levels of luciferase expression compared to *Pm*-regulated cells during exponential growth (Fig 7A). We measured luciferase activity in the uninduced samples and we observed a background expression from *Pm that* was low throughout the experiment. For pUV15tetORm::luciferase we observed an increase in background expression over time (Fig 7B).

Taken together, our results indicate that *Mtb* basal expression mediated by *Pm* is significantly lower than *Ptet* basal expression, under the given conditions. While *Ptet*-regulated constructs result in higher induction, XylS/*Pm* may provide an advantage for experiments where consistent low background expression is of particular importance.

## Discussion

In this study we have successfully introduced a benzoic acid-inducible expression system to mycobacteria, and expanded the repertoire of mycobacterial tools for studying gene function and essentiality. XylS/*Pm* has previously been thoroughly studied and applied in Gram-negatives (reviewed in [6]). Importantly, by adapting *Pm*-regulated expression to mycobacteria, we demonstrate for the first time the great potential of expanding the use of this expression system to non-Gram-negative species.

Upon induction with *m*-toluate, we show a typical 90-fold increase for *Pm*-mediated reporter gene expression in *Msmeg* (Fig 2A), an induction ratio comparable to or higher than the reported ratios of all other published inducible mycobacterial expression systems based on reporter gene assays [15–19, 21, 23]. The exception is the pristamycin-inducible system with a reported 400-fold induction in *M*. *tuberculosis* [21]. We also report a dose-dependent response, with higher concentrations of inducer leading to increased induction ratios (Fig 2A). Although we might reach even greater expression levels by further increasing inducer concentrations, due to a decrease in growth rate in the presence of more than ~1.5 mM *m*-toluate (S1 Fig) we chose to largely avoid concentrations above 1.5 mM. Furthermore, in this study we focused on one of many XylS/*Pm*-inducers known to work in Gram-negative bacteria [45]. Other inducers might compare favorably to *m*-toluate when it comes to both inducibility and toxicity in mycobacteria.

While *Pm*-mediated expression clearly functions in *Mtb*, we are not able to reach the same level of induced expression as in *Msmeg*, and we do not see the same dose-dependent response (Fig 3A). Nevertheless, as in *Msmeg*, we see a time-dependent response, with a peak in expression in late log phase (Fig 3A and 3B). The ~10-fold increase in induction observed using 0.15 mM *m*-toluate is comparable to the 0.15 mM-induction of *Msmeg*, but increasing inducer concentration further does not boost expression in *Mtb*. Mycobacterial drug efflux pumps have been identified in *Mtb* for inducers like tetracycline [46, 47], and we cannot exclude similar active pumping of *m*-toluate out of the cell, resulting in a decreased maximal response to induction. Alternatively, *m*-toluate could be metabolized by mycobacteria, decreasing the concentration of inducer in the cell, as oxidation of benzoate and related substances was reported in early mycobacterial research [48]. Another inducer might thus be superior to *m*-toluate for XylS/*Pm*-induction in *Mtb*.

XylS/*Pm* can be suitable to regulate conditionally essential genes in mycobacteria. We cloned the *Sh ble* gene, which confers zeocin resistance, under the control of *Pm* (pMDX-zeo) in *Msmeg*. Our results demonstrated that induced cells containing pMDX-zeo survived significantly better in the presence of zeocin than uninduced cells, with growth of uninduced cells inhibited by as little as 1 μg/ml zeocin (Fig 5). Although the growth rate of uninduced cells that carry the resistance gene showed inhibition at 1 μg/ml zeocin, cultures did grow, albeit slowly, in the presence of up to 5–10 μg/ml of the antibiotic. In contrast, cells transformed with a vector lacking *Sh ble*, did not grow at 0.5 μg/ml zeocin (results not shown). This observation suggests that the uninduced basal expression of the resistance gene is appreciably low, but might be biologically relevant depending on the gene in question. When we controlled expression of the alanine racemase gene with the *Pm* and *Ptet* promoters, we observed that background expression of the conditionally essential gene was sufficient to maintain a wild type phenotype.

Comparing *Pm*-mediated background expression with the background expression of the widely used tet-inducible *Ptet*-promoter [16], *Pm* in fact drives lower levels of basal expression than *Ptet* both in *Msmeg* and *Mtb* (Figs 6B and 7B). Here, *Ptet* basal expression level (unlike *Pm*) is dependent on active repression of transcription. A codon usage adaption of the Tn*10*-derived TetR for optimal gene regulation in *Mtb* resulted in 3-fold improved repression in *Msmeg* when the repressor was expressed episomally, due to increased TetR steady state levels [31]. It is plausible that the increase in *Ptet*-mediated background expression we observed in stationary phase is due to a decrease in TetR steady state levels during prolonged growth, making the repressor-independent nature of *Pm*-mediated basal levels a likely explanation for the lower background expression seen in XylS/*Pm*. Reverse gene regulation tools, where the addition of the effector molecule turns transcription off instead of on, have been developed to silence genes in mycobacteria [31, 49, 50]. This approach has proved useful to study conditional mutants [50, 51], however, these systems are still dependent on sufficient expression of the repressor to keep the gene expression low. In our system, the consistent low basal expression coupled to titratable, strong induction makes *Pm* an appealing option for regulated gene expression in mycobacteria, and XylS/*Pm* may be a particularly good alternative when a consistent, low background expression is important.

Basal rates of *Pm*-regulated expression were not reduced by addition of atc, which suggests that the XylS production is unlikely to be high enough to drive transcription from the uninduced construct. Instead, the low background expression observed in our experiments is most probably due to leaky transcription from the *Pm* promoter itself initiated at a low rate by the endogenous transcription machinery. Consequently, future work toward even tighter control of *Pm* basal transcription in mycobacteria would include random mutagenesis of the promoter or the 5’ untranslated region, which previously has been shown to improve XylS/*Pm*-mediated regulation in *E*. *coli* [43, 52].

An important aspect of mycobacterial research is the modulation of gene expression during infection. Tet-inducible expression systems are thus far the only tools for induction in macrophage or animal models of mycobacterial infection [15, 16, 18]. The XylS/*Pm*-inducers are weak acids passively diffusing through plasma membranes. Medina *et al*. successfully induced expression of dTomato reporter protein under the control of the *Pm* promoter in *Salmonella* during the infection of HeLa cells, using salicylate as inducer [53]. These results support the potential function of our adapted XylS/*Pm* expression system during mycobacterial infections of macrophages. Benzoic acid is commonly used as a food preservative [54, 55], which together with the fact that benzoic acids can readily diffuse across plasma membranes suggests that inducers of P*m*-mediated expression could be excellent candidates for oral administration in animal models. Thus, XylS/*Pm* has potential to become the second inducible regulator of mycobacterial expression applicable in animal models of tuberculosis.

## Supporting Information

S1 Fig Growth curves in the presence of m-toluate. (A) *Msmeg* and (B) *Mtb* grown in 7H9 medium with increasing concentrations of *m*-toluate. OD_600_ was determined after 2.5, 5, 8, 10 and 24 hours (*Msmeg*), or 4, 10 and 14 days (*Mtb*).
(TIF)

S2 Fig Induced luciferase expression normalized to OD_600_ and RLU. *Msmeg* transformed with pMDX or pMDX-luc was grown to stationary phase, subcultured and grown to OD_600_ 0.05–0.1 before induction by 1.5 mM *m*-toluate (induced) or ethanol carrier (uninduced). Cultures were incubated for 24 hours with shaking at 37°C before luciferase expression was measured. RLU was normalized to (A) OD_600_ or (B) CFU, reaching 110 and 240-fold induction, respectively.
(TIF)

S3 Fig XylS/*Pm* is inducible when integrated in the chromosome. *Msmeg* transformed with pMDXint, pMDXint-luc or pMDX-luc were grown to stationary phase, subcultured and grown to OD_600_ 0.05–0.1 before addition of 1.5 mM *m*-toluate (induced) or ethanol carrier (uninduced). Cultures were incubated for 24 hours with shaking at 30°C before luciferase expression was measured. Results are presented as RLU normalized to OD_600_. Results are representative for three individual experiments.
(TIF)

S4 Fig Induction of *Pm* is robust at both 30 and 37°C. *Msmeg* transformed with pMDX-luc was grown to stationary phase, subcultured and grown to OD_600_ 0.05–0.1 before addition of 0.05, 0.25, 1 or 1.5 mM *m*-toluate (induced) or ethanol carrier (uninduced). Cultures were incubated for 24 hours with shaking at 30 or 37°C before luciferase expression was measured. Results are presented as fold induction of RLU normalized to OD_600_ of induced compared to uninduced samples. The fold inductions of RLUs comparing 30°C and 37°C were obtained in separate luciferase assays. Results are representative for two or more independent experiments.
(TIF)

S5 Fig Comparison of *Ptet* and *Pm*-mediated expression. *Msmeg* containing pTET-zeo or pMDX-zeo was grown to stationary phase, then diluted to OD_600_ 0.005 in the presence or absence of inducer. pMDX-zeo was induced with 1.5 mM *m*-toluate, and pTET-zeo was induced with 200 ng/ml anhydro-tetracycline (atc). The samples were grown in triplicates in micro-plate wells in increasing amounts of zeocin (0, 0.5, 2.5, 5, 7.5, 10, 15, 25, 50, 100, 150, 200, 250 or 500 μg/ml). Growth was monitored for 120 hours using a Bioscreen, shaking at 37°C, registering OD_600_ every other hour. All growth curves represent the average of 3 replicates.
(TIF)

S6 Fig Alanine racemase gene controlled by *Pm* and *Ptet*. *Msmeg* wt, DM22 mutant and DM22 containing pTET-alr or pMDX-hyg-alr were grown to stationary phase then serial diluted and spotted onto agar plates with or without D-alanine.
(TIFF)
