# Supplementary figures and images for: Benzoic Acid-Inducible Gene Expression in Mycobacteria
Source: PLoS One. 2015 Sep 8;10(9):e0134544. doi: 10.1371/journal.pone.0134544 (PMC4562662; doi:10.1371/journal.pone.0134544)

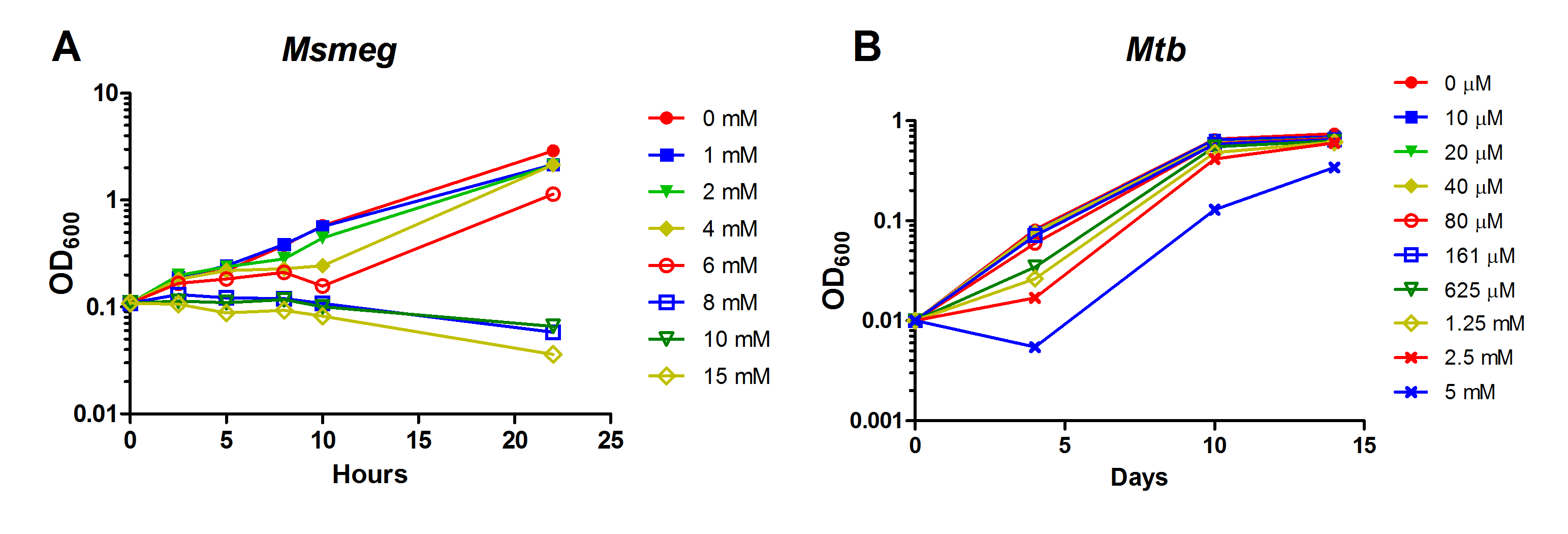

Supplement: S1 Fig — (A) Msmeg and (B) Mtb grown in 7H9 medium with increasing concentrations of m-toluate. OD600 was determined after 2.5, 5, 8, 10 and 24 hours (Msmeg), or 4, 10 and 14 days (Mtb). (TIF) [file pone.0134544.s001.tif]

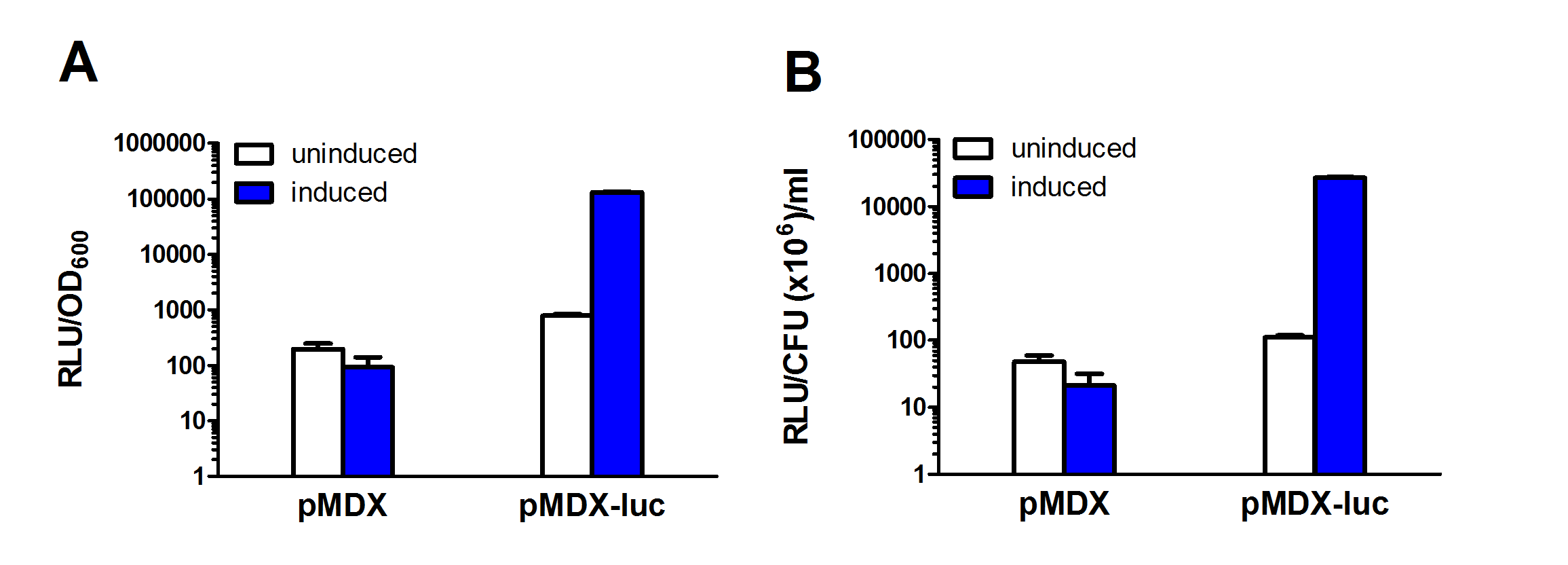

Supplement: S2 Fig — Msmeg transformed with pMDX or pMDX-luc was grown to stationary phase, subcultured and grown to OD600 0.05–0.1 before induction by 1.5 mM m-toluate (induced) or ethanol carrier (uninduced). Cultures were incubated for 24 hours with shaking at 37°C before luciferase expression was measured. RLU was normalized to (A) OD600 or (B) CFU, reaching 110 and 240-fold induction, respectively. (TIF) [file pone.0134544.s002.tif]

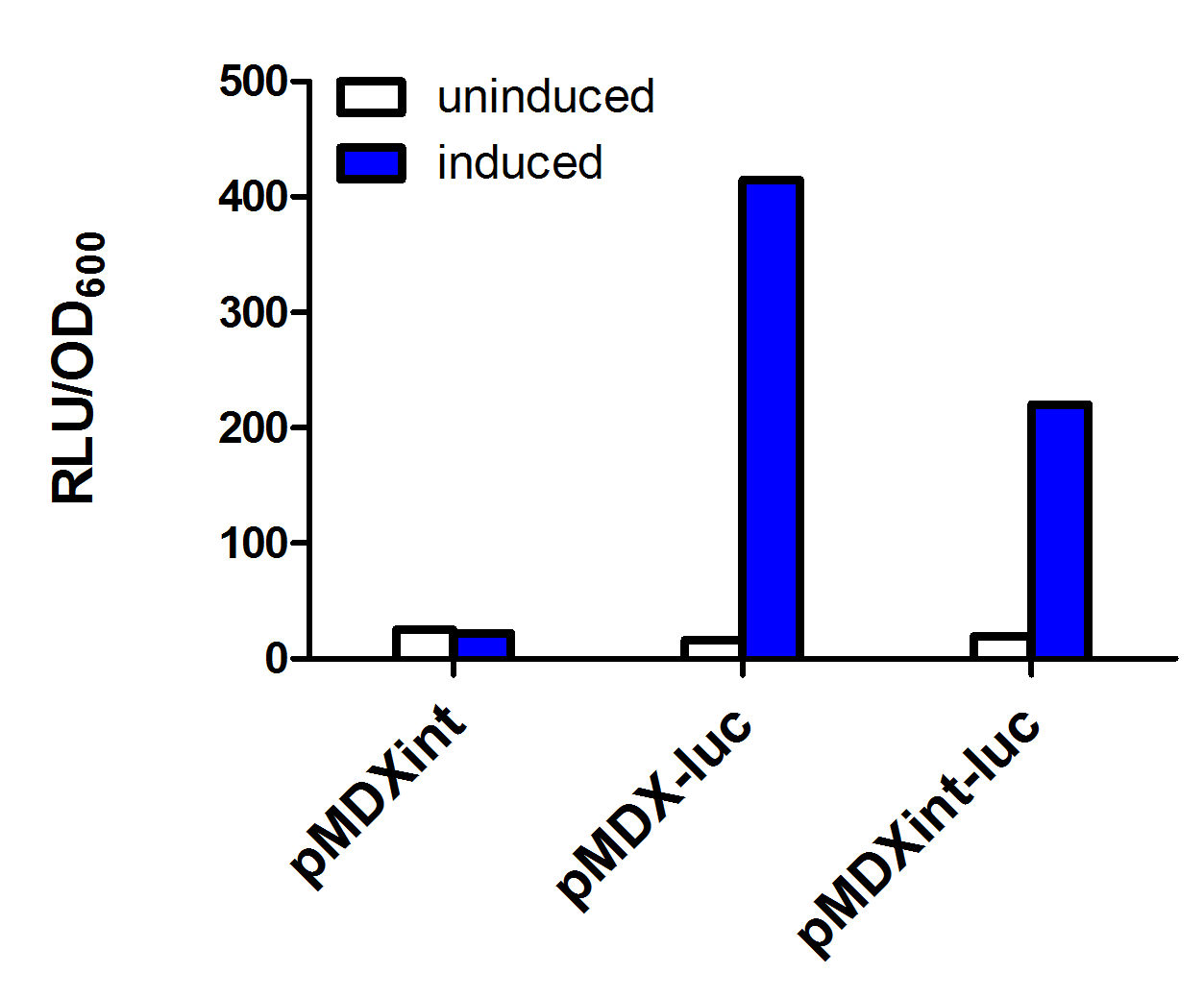

Supplement: S3 Fig — Msmeg transformed with pMDXint, pMDXint-luc or pMDX-luc were grown to stationary phase, subcultured and grown to OD600 0.05–0.1 before addition of 1.5 mM m-toluate (induced) or ethanol carrier (uninduced). Cultures were incubated for 24 hours with shaking at 30°C before luciferase expression was measured. Results are presented as RLU normalized to OD600. Results are representative for three individual experiments. (TIF) [file pone.0134544.s003.tif]

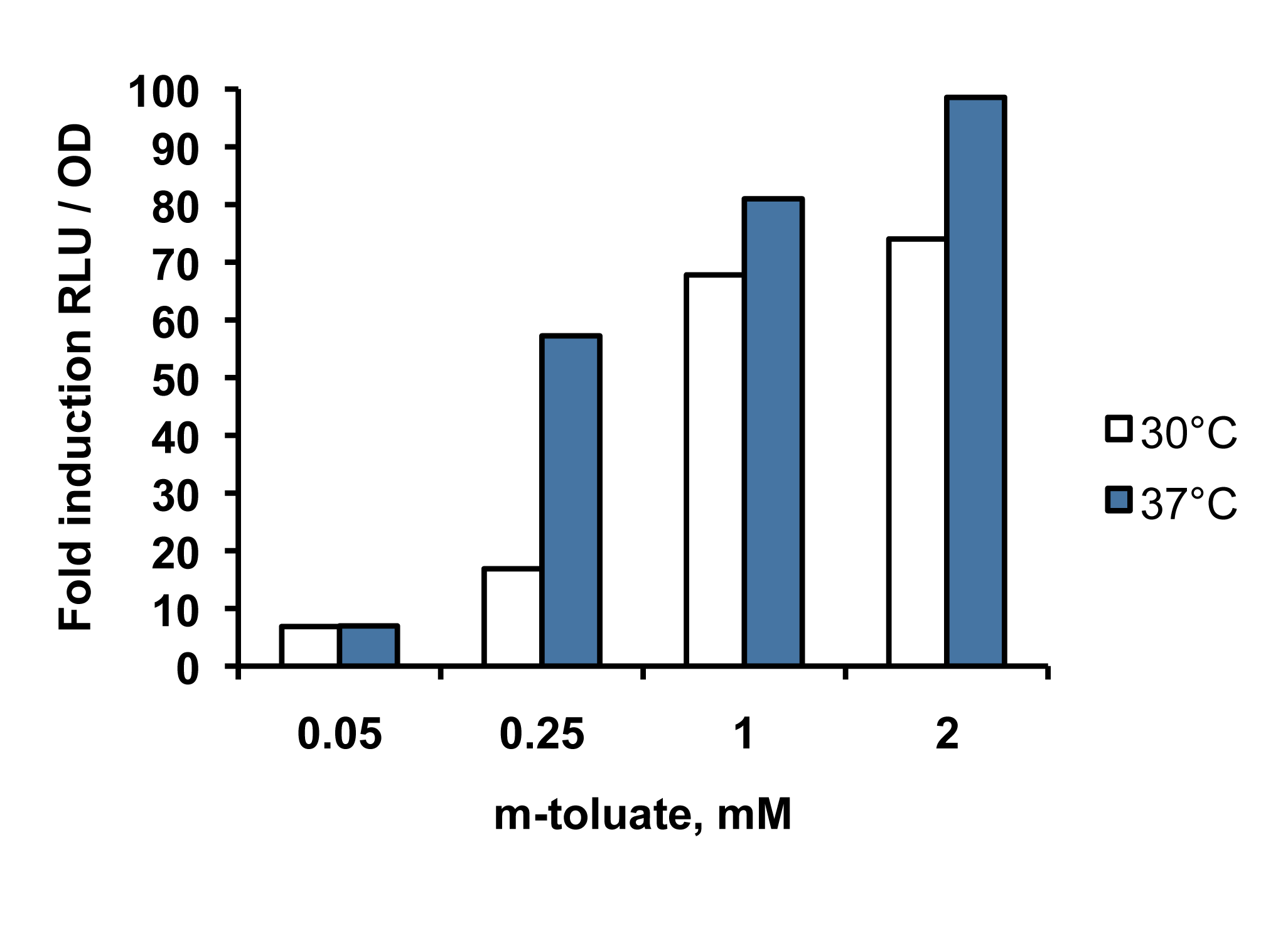

Supplement: S4 Fig — Msmeg transformed with pMDX-luc was grown to stationary phase, subcultured and grown to OD600 0.05–0.1 before addition of 0.05, 0.25, 1 or 1.5 mM m-toluate (induced) or ethanol carrier (uninduced). Cultures were incubated for 24 hours with shaking at 30 or 37°C before luciferase expression was measured. Results are presented as fold induction of RLU normalized to OD600 of induced compared to uninduced samples. The fold inductions of RLUs comparing 30°C and 37°C were obtained in separate luciferase assays. Results are representative for two or more independent experiments. (TIF) [file pone.0134544.s004.tif]

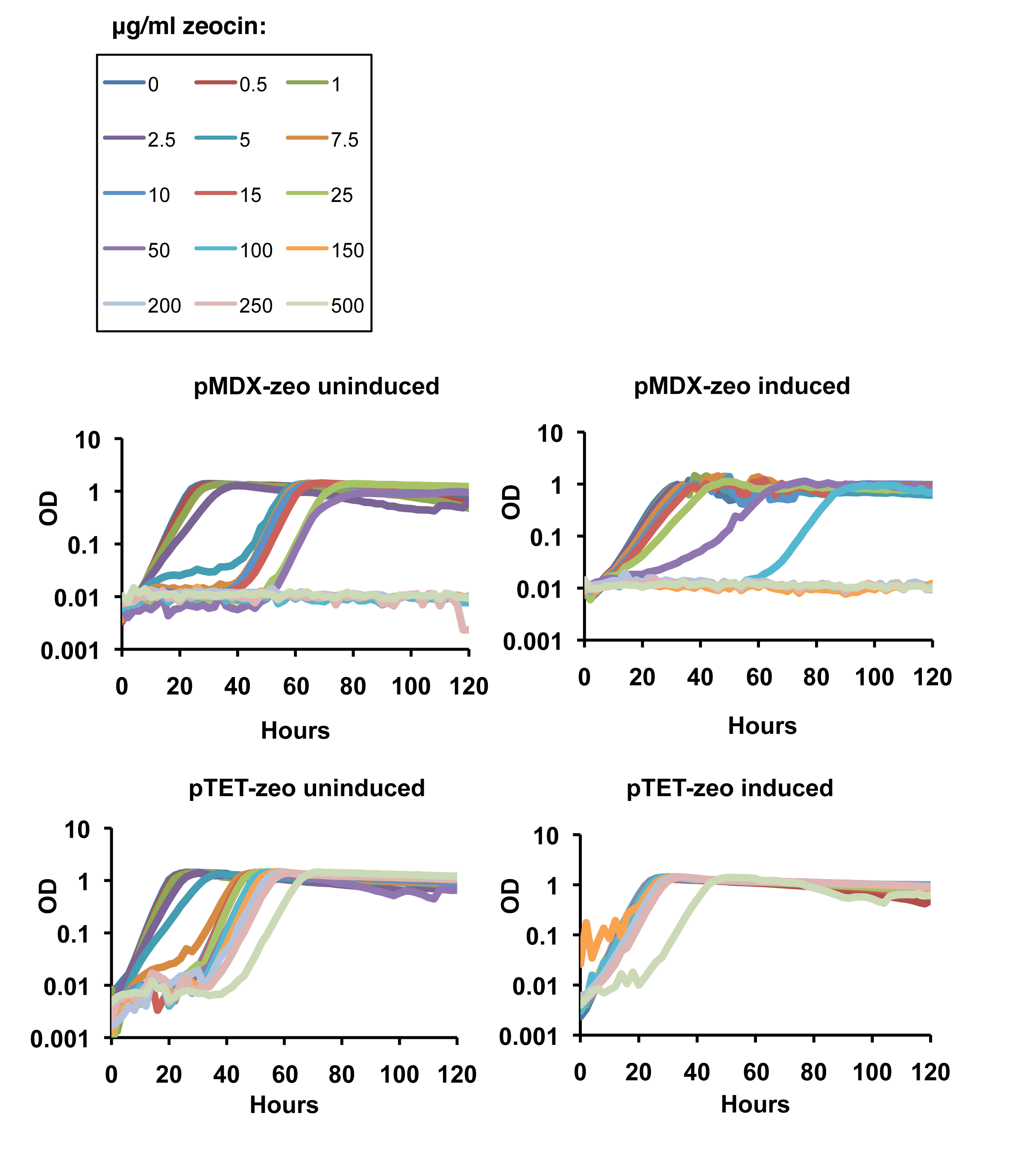

Supplement: S5 Fig — Msmeg containing pTET-zeo or pMDX-zeo was grown to stationary phase, then diluted to OD600 0.005 in the presence or absence of inducer. pMDX-zeo was induced with 1.5 mM m-toluate, and pTET-zeo was induced with 200 ng/ml anhydro-tetracycline (atc). The samples were grown in triplicates in micro-plate wells in increasing amounts of zeocin (0, 0.5, 2.5, 5, 7.5, 10, 15, 25, 50, 100, 150, 200, 250 or 500 μg/ml). Growth was monitored for 120 hours using a Bioscreen, shaking at 37°C, registering OD600 every other hour. All growth curves represent the average of 3 replicates. (TIF) [file pone.0134544.s005.tif]

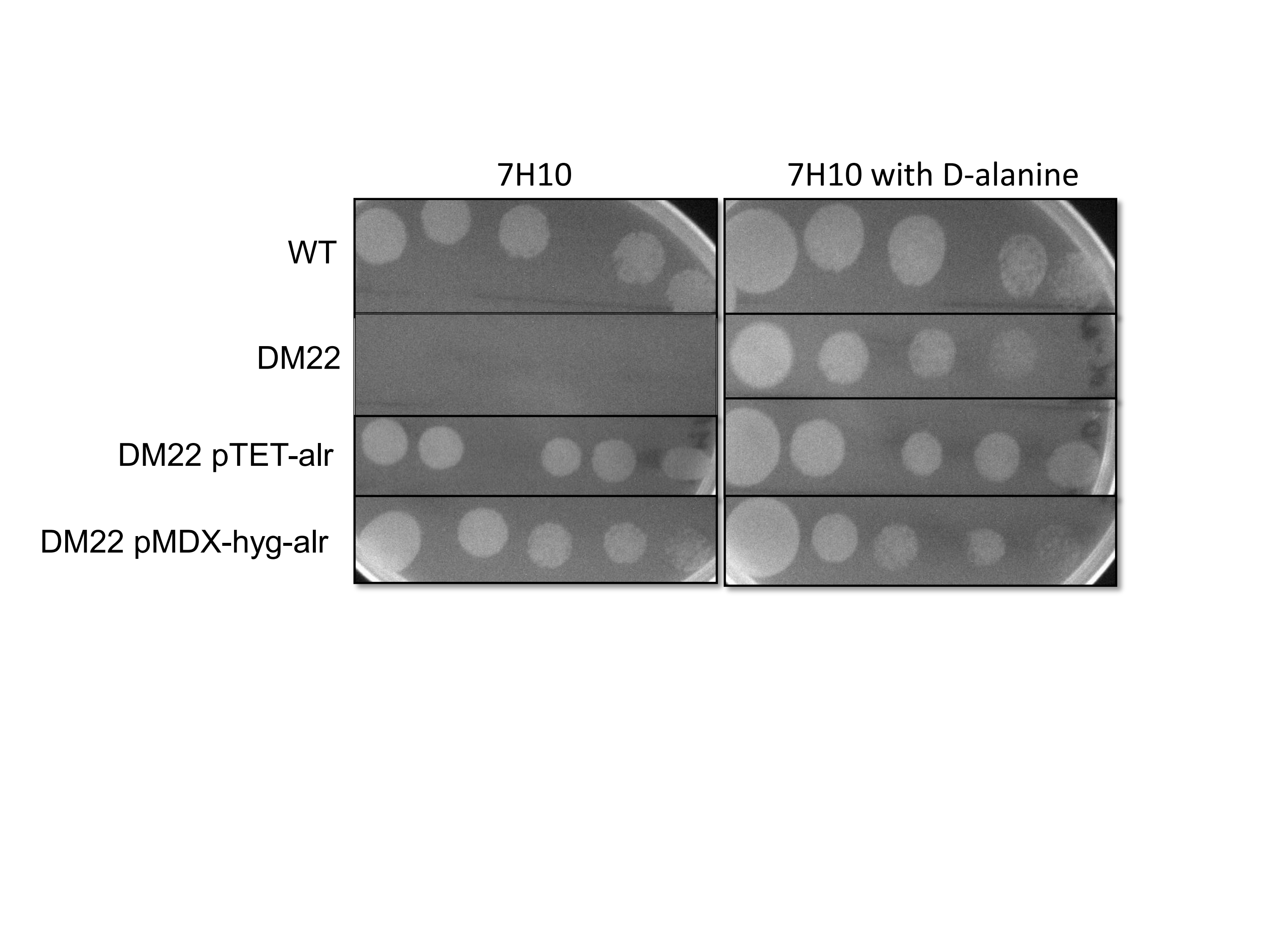

Supplement: S6 Fig — Msmeg wt, DM22 mutant and DM22 containing pTET-alr or pMDX-hyg-alr were grown to stationary phase then serial diluted and spotted onto agar plates with or without D-alanine. (TIFF) [file pone.0134544.s006.tiff]
